# Identification and Validation of ERK5 as a DNA Damage Modulating Drug Target in Glioblastoma

**DOI:** 10.3390/cancers13050944

**Published:** 2021-02-24

**Authors:** Natasha Carmell, Ola Rominiyi, Katie N. Myers, Connor McGarrity-Cottrell, Aurelie Vanderlinden, Nikita Lad, Eva Perroux-David, Sherif F. El-Khamisy, Malee Fernando, Katherine G. Finegan, Stephen Brown, Spencer J. Collis

**Affiliations:** 1 Weston Park Cancer Centre, Department of Oncology & Metabolism, The University of Sheffield Medical School, Sheffield S10 2SJ, UK; NCarmell1@sheffield.ac.uk (N.C.); o.rominiyi@sheffield.ac.uk (O.R.); k.myers@sheffield.ac.uk (K.N.M.); clmcgarritycottrell1@sheffield.ac.uk (C.M.-C.); avanderlindendibekeme1@sheffield.ac.uk (A.V.); Nikita.lad2019@my.ntu.ac.uk (N.L.); eva.perroux-david@estbb.ucly.fr (E.P.-D.); 2 Department of Neurosurgery, Royal Hallamshire Hospital, Sheffield Teaching Hospitals NHS Foundation Trust, Sheffield S10 2JF, UK; 3 Sheffield Institute for Nucleic Acids (SInFoNiA) and the Healthy Lifespan Institute, University of Sheffield, Sheffield S10 2TN, UK; s.el-khamisy@sheffield.ac.uk; 4 Institute of Cancer Therapeutics, University of Bradford, Bradford BD7 1DP, UK; 5 Department of Histopathology, Royal Hallamshire Hospital, Sheffield Teaching Hospitals NHS Foundation Trust, Sheffield S10 2TN, UK; malee.fernando@nhs.net; 6 Faculty of Biology Medicine and Health, University of Manchester, Manchester M13 9PL, UK; K.G.Finegan@manchester.ac.uk; 7 Department of Biomedical Science, The Sheffield RNAi Screening Facility, The University of Sheffield, Sheffield S10 2TN, UK; stephen.brown@sheffield.ac.uk

**Keywords:** ERK5, MAPK7, glioblastoma, temozolomide, DNA damage, sensitisation

## Abstract

**Simple Summary:**

Glioblastomas are high-grade brain tumours and are the most common form of malignancy arising in the brain. Patient survival has improved little over the last 40 years, highlighting an urgent unmet need for more effective treatments for these tumours. Current standard-of-care treatment involves surgical removal of as much of the tumour as possible followed by a course of chemo-/radiotherapy. The main chemotherapeutic drug used is called temozolomide, however even with this treatment regimen, the average patient survival following diagnosis is around 15 months. We have identified a protein called ERK5 which is present at higher levels in these high-grade brain tumours compared to normal brain tissue, and which is also associated with resistance to temozolomide and poor patient survival. Additionally, we show that targeting ERK5 in brain tumour cells can improve the effectiveness of temozolomide in killing these tumour cells and offers potential much-needed future clinical benefit to patients diagnosed with glioblastoma.

**Abstract:**

Brain tumours kill more children and adults under 40 than any other cancer, with approximately half of primary brain tumours being diagnosed as high-grade malignancies known as glioblastomas. Despite de-bulking surgery combined with chemo-/radiotherapy regimens, the mean survival for these patients is only around 15 months, with less than 10% surviving over 5 years. This dismal prognosis highlights the urgent need to develop novel agents to improve the treatment of these tumours. To address this need, we carried out a human kinome siRNA screen to identify potential drug targets that augment the effectiveness of temozolomide (TMZ)—the standard-of-care chemotherapeutic agent used to treat glioblastoma. From this we identified ERK5/MAPK7, which we subsequently validated using a range of siRNA and small molecule inhibitors within a panel of glioma cells. Mechanistically, we find that ERK5 promotes efficient repair of TMZ-induced DNA lesions to confer cell survival and clonogenic capacity. Finally, using several glioblastoma patient cohorts we provide target validation data for ERK5 as a novel drug target, revealing that heightened ERK5 expression at both the mRNA and protein level is associated with increased tumour grade and poorer patient survival. Collectively, these findings provide a foundation to develop clinically effective ERK5 targeting strategies in glioblastomas and establish much-needed enhancement of the therapeutic repertoire used to treat this currently incurable disease.

## 1. Introduction

Glioblastomas (also known as glioblastoma multiforme; GBM) are the most frequently diagnosed primary brain tumour in adults and are responsible for the greatest reduction in life expectancy of any cancer, accounting for around 200,000 global deaths each year [1]. High-grade gliomas are characterised by treatment resistance and rapid disease recurrence, making them an incurable cancer [2]. Current therapeutic interventions for these tumours consists of debulking surgical resection followed by radio-/chemotherapy regimens. The mainstay chemotherapy is the DNA alkylating agent temozolomide (TMZ), which was clinically approved in 2007 due to significant improvements in patient survival of around 2.5 months compared with radiotherapy alone [3]. However, the median survival for these patients remains poor at around 14.6 months, with less than 10% of patients surviving more than 5 years [4]. As such, gliomas have been recognised as a cancer of unmet need that urgently requires new therapeutic intervention and approaches to help improve treatment and patient survival [5].

In order to identify putative new therapeutic targets that could potentially synergise with current TMZ chemotherapy treatments, we carried out a human kinome targeted RNAi screen in inherently TMZ-resistant glioma cells. From this, we identified several kinases whose depletion led to significantly increased sensitivity to the cytotoxic effects of temozolomide. Of particular interest within the identified hits was extracellular signal-regulated kinase 5; ERK5 (also known as MAPK7 or BMK-1; UniProtKB: Q13164), which is a relatively understudied member of the MAP kinase family. Interestingly, ERK5 has recently gained prominence in the oncology field as a potential novel drug target due to dysfunction within several tumour types and a strong association with therapy resistance [6,7,8,9,10,11]. Indeed, several groups as well as the pharmaceutical industry are actively developing new specific inhibitors towards ERK5 for potential use within the oncology setting [12,13,14,15].

Using a range of glioma cell lines, RNAi chemistries and ERK5 small molecule inhibitors, we report here that ERK5 promotes glioma cell resistance to TMZ. Furthermore, we show that disruption to ERK5 in combination with TMZ treatments leads to increased amounts of DNA damage and cell death through mitotic associated mechanisms. Finally, using online mRNA databases and a range of glioma tumour tissue samples from several archived patient cohorts, we carried out the first comprehensive appraisal of ERK5 protein expression in adult gliomas, revealing that high levels of endogenous ERK5 expression are associated with higher grade tumours and poorer survival. Collectively, our data identify and validate ERK5 as a novel therapeutic target for high-grade adult gliomas.

## 2. Results

### 2.1. Identification and Validation of ERK5 as a Novel Temozolomide Resistance Factor in GBM

In order to identify potential factors that promote resistance to temozolomide (TMZ), the standard of care chemotherapy for patients with glioblastoma, we carried out a kinase targeted RNAi screen in inherently TMZ resistant T98G cells to identify kinases whose RNAi-mediated targeting led to increase TMZ sensitivity (Figure S1A). We chose to target human kinases since this family of proteins are overtly dysregulated and subsequently studied in cancer [16]. This decision was also based on the approved clinical use of multiple kinase targeting drugs for oncology, as well as the numerous readily available preclinical kinase inhibitors that have been developed and/or are in current oncology-based clinical trials [17]. As such, we envisaged that hits identified in such screens were most able to be rapidly progressed into preclinical and clinical studies, which is a much-needed criteria considering the lack of sufficient clinical progress in treating high-grade gliomas. 

**Figure 1:**
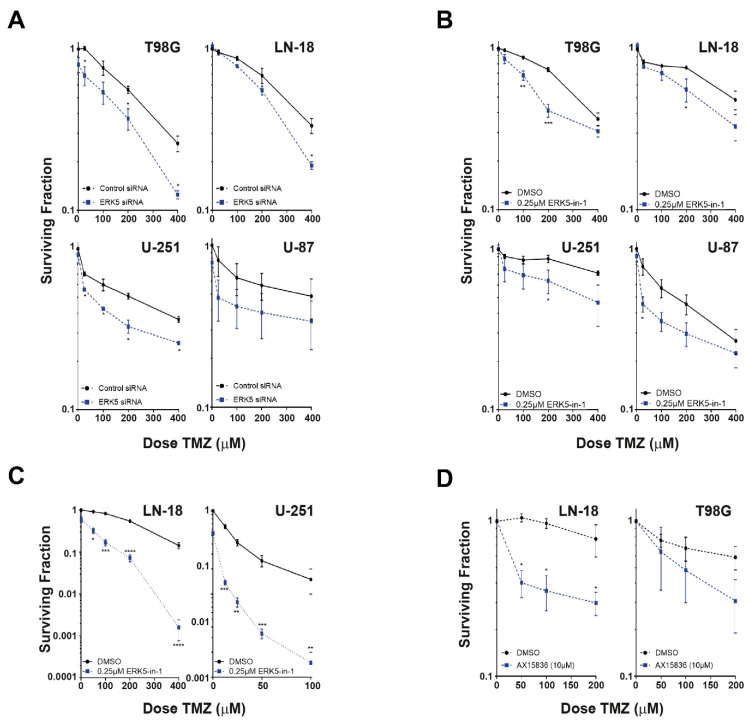
Identification and validation of ERK5 as a novel temozolomide (TMZ) resistance factor in GBM. (**A**): TMZ cytotoxicity as assessed by MTT assays in the indicated glioblastoma cell lines transfected with either control or ERK5 siPOOLs. (**B**): TMZ cytotoxicity as assessed by MTT assays in the indicated glioblastoma cell lines pretreated with either DMSO or 250 nM of the ERK5-in-1 small molecule ERK5 inhibitor. (**C**): TMZ cytotoxicity as assessed by clonogenic survival assays in the respective MGMT +ve LN18 and MGMT −ve U-251 glioblastoma cells pretreated with either DMSO or 250 nM of the ERK5-in-1 small molecule ERK5 inhibitor. (**D**): TMZ cytotoxicity as assessed by clonogenic survival assays in the indicated glioblastoma cell lines pretreated with either DMSO or 10 μM of the AX15836 small molecule ERK5 inhibitor. All data shown are the means derived from at least three independent experimental repeats together with their associated standard errors. Statistical significance was calculated using the nonparametric Mann–Whitney U-test: ns = not significant, * = *p* < 0.05, ** = *p* < 0.01, *** = *p* < 0.001, and **** = *p* < 0.0001 comparing the indicated treatment to DMSO controls or to another indicated treatment cell population.

Of particular interest amongst the putative hits identified within this screen was ERK5 (also known as MAPK7 and MEK5), as it has recently been identified as a potential oncogenic factor in several other cancers [6,7,8,9,10,11,18,19,20], and several potential therapeutic small molecule inhibitors have recently been developed [12,13,14,15,21]. To further validate ERK5 as promoting TMZ resistance in glioma cells and to discount potential off-target RNAi effects, we used siPOOLs consisting of 30 individual low nM siRNA that have been shown to give effective knockdown at low total nM concentrations (Figure S1B) with reduced off-target effects [22]. In agreement with our RNAi screen, knockdown of ERK5 in T98G cells with 10 nM ERK5 siPOOLs led to increased TMZ sensitivity (Figure 1A). Furthermore, siPOOL mediated knockdown of ERK5 also sensitised resistant LN18 cells to TMZ as well as the more TMZ sensitive U87 and U-251 cells (Figure 1A), demonstrating that targeting of ERK5 could potentially potentiate TMZ cytotoxicity in both MGMT+ and MGMT− glioma cell backgrounds (Figure S1C). This is important from a potential clinical benefit point of view given that around half of these tumours are MGMT +ve and around half are MGMT −ve.

To further validate targeting ERK5 as a valid TMZ sensitising strategy, we used an alternative chemical approach to inhibit ERK5 activity (Figure S1D). Treatment of T98G, LN18, U-251, and U87 cells with the small molecule inhibitor ERK5-in-1, which is potent and specific to ERK5 within the ERK family of kinases [23], sensitised them to TMZ (Figure 1B,C). Similar results were obtained with the historic ERK5i XMD8-92 (Figure S1E). Importantly, pretreatment of TMZ resistant glioma cells with the more recently developed AX15836 compound (XMD17-109), which has a different chemistry and MoA to other ERK5i such as ERK5-in-1 and XMD8-92 [24], also conferred increased TMZ sensitivity in resistant glioma cells. Collectively, these data identify and validated ERK5 as a bone fide strategy to sensitise glioma cells to the current standard of care chemotherapeutic agent TMZ.

### 2.2. ERK5 Inhibition in Combination with TMZ Increases Cellular Levels of DNA Damage

**Figure 2:**
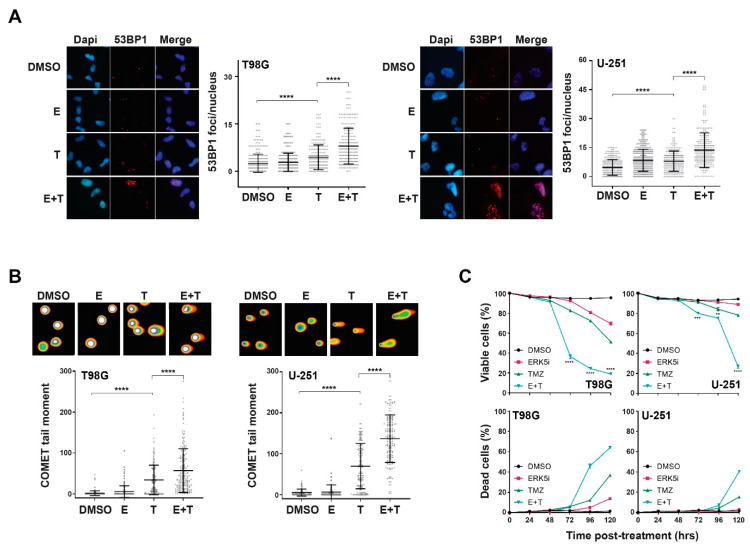
ERK5 promotes efficient repair of TMZ-induced DNA damage and cell survival. (**A**): Representative immunofluorescence images (left panels) and associated scoring (right panels) of 53BP1 nuclear foci in T98G and U251 cells 24 h following treatment with either DMSO, 250 nM of ERK5-in-1 (E), 200 μM TMZ (T), or a combination of the two treatments (E + T). (**B**): Representative immunofluorescence images and scoring of DNA COMETs in T98G and U-251 cells 24 h following the indicated treatments. (**C**): Percentage of viable (top) and dead (bottom) T98G and U-251 cells assessed by annexin V/propidium dual staining at the indicated times post treatment with DMSO, 250 nM ERK5-in-1 (E), 200 μM TMZ (T) or a combination of the two (E + T). All data shown are either individual data points derived from at least three independent experimental repeats (scatter plots) or means derived from three independent experimental repeats (line graphs) together with their associated standard errors. Statistical significance was calculated using the nonparametric Mann–Whitney U-test (A and B) or Kruskal–Wallis H test (C): ns = not significant, ** = *p* < 0.01, *** = *p* < 0.001, and **** = *p* < 0.0001 comparing the indicated treatment to DMSO controls or to another indicated treatment cell population.

We next sought to determine the mechanism(s) behind the increase sensitivity to TMZ following disruption to ERK5 function. Treatment of cells with TMZ did not induce ERK5 activation (Figure S2A), and importantly, inhibition of ERK5 did not lead to reduced MGMT levels, or a statistically significant increase in O6-methylguanine production when combined with TMZ, two mechanisms that could account for the increased TMZ sensitivity phenotype (Figure S2B,C, respectively). However, we found that treatment of either MGMT +ve or MGMT −ve glioma cells with TMZ in combination with ERK5 inhibition led to a significant increase in DNA damage as assessed by both 53BP1 foci prevalence (an established marker of DNA double-strand breaks [25,26]) and direct visualisation of DNA damage by COMET assay (Figure 2A,B and Figure S2D), which was accompanied by a significant increase in apoptotic cells and reduced cell viability (Figure 2C). These data therefore suggest that increased amounts of DNA damage accumulate in ERK5 inhibited cells following TMZ treatment, leading to increased levels of apoptotic cell death and reduced cell survival/clonogenicity.

**Figure 3:**
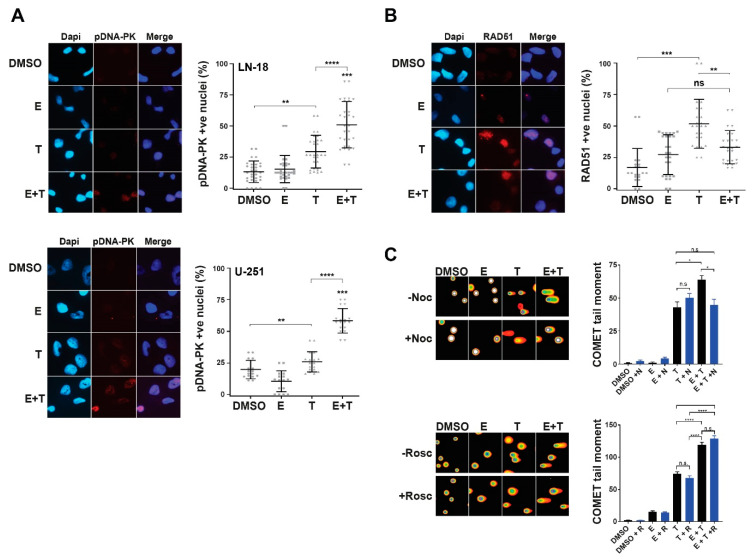
ERK5 inhibition leads to TMZ-induced activation of NHEJ and increased post-mitotic DNA damage. (**A**): Representative immunofluorescence images (left panels) and associated scoring (right panels) of pDNA-PKcs (Ser2056) nuclear foci in LN18 and U-251 cells 24 h following treatment with either DMSO, 250 nM of ERK5-in-1 (E), 200 μM TMZ (T), or a combination of the two treatments (E + T). (**B**): Representative immunofluorescence images (left panels) and associated scoring (right panels) of RAD51 nuclear foci in LN18 cells 24 h following treatment with either DMSO, 250 nM of ERK5-in-1 (E), 200 μM TMZ (T), or a combination of the two treatments (E + T). (**C**): Representative immunofluorescence images (left panels) and associated scoring (right panels) of DNA COMETs in LN18 cells 24 h following treatment with either DMSO, 250 nM of ERK5-in-1 (E), 200 μM TMZ (T), or a combination of the two treatments (E + T) with or without treatment of either 10 μM roscovitine for 4 h (upper dataset) or 1 ng/mL nocodazole for 24 h (lower dataset). All data shown are either individual data points derived from at least three independent experimental repeats (scatter plots) or means derived from three independent experimental repeats (bar graphs) together with their associated standard errors. Statistical significance was calculated using the nonparametric Mann–Whitney U-test: ns = not significant, * = *p* < 0.05, ** = *p* < 0.01, *** = *p* < 0.001, and **** = *p* < 0.0001 comparing the indicated treatment to DMSO controls or to another indicated treatment cell population.

In order to explore the nature of the TMZ-induced DNA damage and subsequent DNA damage response mechanisms in ERK5 inhibited cells following TMZ treatment, we assessed phosphorylated DNA-PKcs (Ser2056) and RAD51 nuclear foci as respective established markers of DNA replication-associated non-homologous end-joining (NHEJ) and homologous recombination (HR) DNA repair mechanisms [27,28]. Combination treatments of TMZ with ERK5i induced significant increases in cells exhibiting pDNA-PK nuclear foci over each treatment alone (Figure 3A), which was not the case for RAD51 foci formation (Figure 3B); a known contributing factor to cellular resistance to TMZ in glioma cells [29]. This suggests that TMZ-induced DNA lesions in ERK5i inhibited cells are inappropriately activating NHEJ post S-phase rather than higher fidelity HR DNA repair processes. Indeed, cells treated with TMZ in combination with ERK5i exhibit reduced levels of RAD51 foci (Figure 3B). This is also consistent with an increased frequency of micronuclei observed in ERK5i TMZ-treated cells (Figure S2E). 

To explore this further, we inhibited either S-phase entry with the pan-CDK inhibitor roscovitine [30,31], or mitotic progression with the microtubule polymerisation inhibitor nocodazole [32,33] and assessed DNA damage in these cells following TMZ and ERK5i treatment. Consistent with our previous findings (Figure 2), combination treatments of TMZ and ERK5i led to increased levels of DNA damage above that observed in cells treated with TMZ alone (Figure 3C). Importantly, this increased DNA damage was alleviated by nocodazole but not roscovitine (Figure 3C). Combined with our other findings, these data suggest that inhibition of ERK5 causes a reduction in HR-mediated DNA repair processes, resulting in an over-reliance on more error-prone NHEJ, leading to post-mitotic induced DNA damage and cell death; a recently identified phenomenon in genetically unstable cell populations [34,35,36]. Use of ERK5i in combination with standard of care TMZ treatments might therefore offer a potential new therapeutic intervention strategy to treat traditionally TMZ resistant gliomas.

### 2.3. Target Validation of ERK5 in Glioma Patient Cohorts

**Figure 4:**
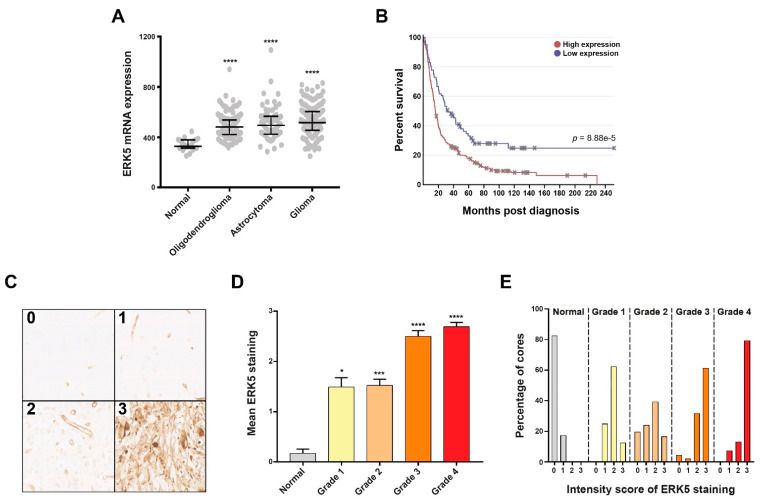
Primary brain tumours exhibit heightened ERK5 expression which is associated with tumour grade and poor patient survival. (**A**): Scatter plot showing relative ERK5 mRNA expression in the indicated tissue samples derived from the REMBRANDT online brain tumour repository of 524 samples. (**B**): Kaplan–Meier survival curves for brain tumour patients within the REMBRANDT repository differentiated for either low or high relative ERK5 mRNA expression along with the associated Chi-squared significance. (**C**): Representative bright field images of immunohistochemical staining showing the indicated graded intensity of ERK5 protein expression/staining of FFPE brain tumour tissue sections from the Sheffield Royal Hallamshire Hospital cohort. (**D**): Average intensity scoring of ERK5 protein staining within the indicated tumour grade across the 190 samples scored, which consisted of 28 normal brain, 16 grade 1, 64 grade 2, 31 grade 3, and 51 grade 4 gliomas. (**E**): Breakdown of the percentage of FFPE cores exhibiting the indicated staining intensity scores within normal brain samples and each tumour grade for the patient cohorts shown in panel D. Statistical significance was calculated using the Kruskal–Wallis H-test: * = *p* < 0.05, *** = *p* < 0.001, and **** = *p* < 0.0001 comparing the indicated tumour grade cohort to the normal brain cohort.

To further support the potential use of ERKi as a therapeutic strategy for glioblastoma, we assessed the expression of ERK5 in glioma patient cohorts to provide target validation data to support this strategy. Analysis of the REpository for Molecular BRAin Neoplasia DaTa (REMBRANT) dataset [37] revealed that endogenous ERK5 mRNA expression is significantly increased in all grades of brain tumours compared with normal brain tissue (Figure 4A), and that increased ERK5 mRNA expression is associated with worse patient survival rates (Figure 4B).

In order to determine if differential ERK5 expression within brain tumours is also present at the protein level, we optimized ERK5 antibody staining for use on archived FFPE sections (Figure S2F). We subsequently stained a total of 190 normal and brain tumour FFPE cores present on both commercially sourced tissue microarrays and from archived material obtained from the Histopathology Department at Sheffield’s Royal Hallamshire Hospital for ERK5 expression, and established a staining scoring protocol similar to one we have previously used for glioma samples [38] (Figure 4C). Within these patient cohorts, mean endogenous ERK5 protein expression was significantly increased in all grades of brain tumours compared with normal brain tissue (Figure 4C), with an interestingly high percentage (≈60%) of grade 1 tumours exhibiting low levels of ERK5 staining/expression. Furthermore, grade 3 and grade 4 tumours exhibited a greater proportion of tissues with the highest levels of ERK5 expression (Figure 4C). These findings are consistent with recent studies that have identified a key role for ERK5 dysregulation in other cancers [6,7,8,9,10,11,19,20,39] and provides a compelling proof-of-concept rationale, together with our other data, to target ERK5 in high-grade brain tumours as means to augment the effectiveness of current temozolomide-based therapeutic strategies. Collectively, these data validate ERK5 as a credible therapeutic target in glioblastomas, which might portend some level of tumour-selectivity over normal healthy brain tissue.

## 3. Discussion

We report here the identification and validation of ERK5 as a potential new therapeutic drug target in high-grade adult brain tumours to augment standard-of-care temozolomide treatment. Traditionally, the study of the MEK family of kinases in cancer has focused on MEK1 and MEK2, with several MEK1/2 inhibitors approved for the treatment of lung cancer and melanoma [40]. However, over the last several years studies focused on understanding the biological functions of ERK5 have revealed several functional roles in cancer biology [6,7,20,39], which has highlighted ERK5 as an emerging new oncology drug target [8,10]. 

To our knowledge, this is the first report of targeting ERK5 to augment the effectiveness of temozolomide cytotoxicity. However, there has been at least one previously reported screen by Svilar et al. to identify factors that modulate cellular responses to alkylation damage and cytotoxicity induced by temozolomide in glioma cells [41]. Akin to our studies, Svilar et al. used TMZ-resistant T98G cells but a larger “druggable” target siRNA library that incorporates the human kinome. They then cross-referenced hits with previous bacterial and yeast alkylation sensitivity screens which mainly identified DNA glycosylases that function within the base excision repair pathways involved in alkylation damage removal, as well as more surprisingly, enzymes involved in ubiquitin and methylation protein post-translational modification [41]. Interestingly, members of the ubiquitylation system were also identified in a previous cell viability siRNA screen carried out in T98G cells [42]. It may, therefore, be that these cells somewhat rely on an intact ubiquitylation system to support sustained cell viability in vitro.

It is relevant to note that for their screen, Svilar et al. used a 1 mM dose of TMZ, which in our experience can lead to over 90% cytotoxicity in T98G cells (data not shown), whereas we used a dose of 50 μM that generally only leads to between 10% and 20% cytotoxicity (Figure 1). However, such high levels of TMZ-induced cytotoxicity appear to have been mitigated within the Svilar screen by use of a much shorter 48 h TMZ incubation time (reducing cytotoxicity to ≈10%), compared with a 5-day post-TMZ incubation time used within our screen. We purposely chose a longer post-TMZ incubation time as it is well-known that the cytotoxic effects of alkylation-induced DNA damage can take several rounds of DNA replication to become apparent, particularly in the MTT cytotoxicity and clonogenic survival (10–14 days) assays we used. These dosage and post-treatment timing differences might therefore explain why ERK5 was not identified as a hit within the screen reported by Svilar et al. 

Consistent with our findings, recent work from others has highlighted a potential role for ERK5 in prostate cancer cell responses to ionising radiation (IR) and etoposide through a similar impact on NHEJ DNA repair mechanisms that we see in glioma cells [18]. Additionally, recent findings in lung cancer cells have highlighted a potential role of ERK5 in efficient activation of DNA damage response proteins to promote resistance to radiation-induced DNA damage [6]. Preliminary studies we have carried out in established GBM cells do not show a similar increased IR sensitivity through targeting of ERK5 (data not shown), however, further studies focused on radiation treatments alone or in combination with TMZ in a range of GBM cell models, including primary glioma cell models would be interesting to explore in the future given these findings. This is something that we are actively pursuing following on from these studies. Taken together, these data support a previously unappreciated role for ERK5 in cellular responses to DNA damage, outside of its canonical role in shear stress signalling.

In addition to identifying ERK5 as a potential novel drug target to augment TMZ effectiveness in glioblastoma treatment regimes, to our knowledge, we also provide the first target validation data for ERK5 in glioblastoma by directly assessing ERK5 mRNA and protein expression within glioblastoma patient cohorts. These analyses reveal that heightened ERK5 expression is associated with tumour grade and poor overall patient survival. The mechanism behind this differential expression is currently not known, although given that the vast majority of the grade 4 tumours will be IDH WT, it seems unlikely that this is due to genetic/epigenetic reprogramming associated with IDH mutation. Indeed, analysis of over 5900 glioma samples within the cBioPortal database did not reveal any relationship between IDH mutation status and ERK5 expression (Figure S2G). Consistent with our findings and a potential role for ERK5 in various aspects glioma tumour biology, a previous study identified microRNA mediated repression of ERK5 as a possible mechanism to suppress glioma tumour cell growth within in vivo xenograft models through downregulation of epithelial–mesenchymal transition processes [20]. Given this and the noted high frequency of increased low-level ERK5 expression over normal brain tissue (Figure 4E), it would suggest that dysregulation of ERK5 within both early and late stage brain tumours may have a functional impact on tumour development and progression. Importantly, differential ERK5 expression within these low grade tumours might offer a potential therapeutic strategy for these notoriously difficult to treat cancers. Consistent with the notion that ERK5 may have important biological functions in both early and late stage brain tumours, ERK5 was recently identified as an important regulator of diffuse intrinsic pontine glioma (DIPG) cell growth within orthotopic xenograft models, through stabilisation of the proto-oncogene transcription facto MYC [39]. Like glioblastoma, DIPG are particularly aggressive brain tumours, but are associated with paediatric patients rather than adults. Given this and our findings presented here, it would therefore be interesting to assesses ERK5 expression, activity and function within a range of paediatric high-grade brain tumours as well as a comprehensive assessment of ERK5 targeting on TMZ and IR sensitivity in additional clinically relevant adult and paediatric high-grade brain tumour models. In this regard, we have recently initiated studies looking at ERK5 targeting in proven clinically relevant 3D primary glioma stem cell models [43] and as well as models of residual post-surgical disease [44]. 

## 4. Materials and Methods 

### 4.1. Cells and Cell Culture

LN18, T98G and U87 cells were purchased from American Type Culture Collection (ATCC). U251 cells were kindly provided by Professor Susan Short (Leeds Institute of Cancer and Pathology). For ERK5i treatments, stocks were maintained at appropriate concentrations in DMSO and further diluted in DMSO to working stocks before being added to the media of cell cultures to give final treatment concentrations. Cell cultures were pretreated with ERK5i 60–90 min prior to addition of TMZ to the cell culture media. DMSO alone was used as a vehicle control and the final DMSO concentration on cells was 0.2% or less to not negatively affect cell growth/viability. Appropriate concentrations of Roscovitine and Nocadozole were added to the cell media, with the 24 h Nocodazole treatments leading to an enrichment of G2/M cells to 70–80% of the cell population.

### 4.2. RNAi Screening

Optimised numbers of T98G cells (z-prime ≥ 0.5) were reverse transfected in 384 plates using RNAiMAX and 20 nM each 3× siRNA pool targeting one of 710 human kinases (derived from the Ambion Silencer Druggable Genome siRNA Library Version 1.1), negative (six individual nontargeting siRNA) or positive control O6-methylguanine methyltransferase (MGMT) siRNA. MGMT was selected as the positive control for this screen as it is the detoxifying enzyme that removes the TMZ-induced methylguanine DNA adduct from DNA which is the most cytotoxic lesion generated by this alkylating agent. Following a 48-h incubation, 50 μM TMZ was added to each well for 5 days. Cells were then washed in PBS using a ELx405 Select Deep Well Washer and then fixed and stained in 5 µg Hoescht/mL in 4% PFA for 20 min before final PBS washing. Plates were then sealed using a PlateLov Velocity 11 and imaged on a Molecular Devices ImageXpress Micro high content microscope using a Multi Wavelength Cell Scoring application on MetaXpress (v5.3) to analyse images. The whole screen was carried out three independent times (biological repeats), with each experimental repeat containing three siRNA replicates per 384-well plate. Potential hits were those that displayed relatively nontoxic activity alone (z-score > −2) but significantly decreased cell viability combined with TMZ (z-score of <−2.2 cut-off).

### 4.3. Western Blotting

Cell lysates were generated as previously described [38,45]. Protein samples were separated on NuPAGE 4–12% Bis-Tris gradient gels and subsequently transferred to PVDF membranes, which were first blocked for 1 h in 5% milk/PBS-T before being incubated with primary antibodies overnight at 4 °C. After three washes in PBS-T, membranes were incubated with secondary antibodies conjugated to HRP at a concentration of 1:1000. Membranes were again washed three times in PBS-T and then visualised using Pierce ECL Western blotting substrate and developed using medical X-ray film and a Konica SRX 101A Processor. HRP-linked secondary antibodies used were goat anti-mouse or swine anti-rabbit IgG at 1:1000 (Dako P0447 and P0399, respectively).

### 4.4. Immunofluorescence and COMET Assays

Depending on the cell line, between 20,000 and 30,000 cells were seeded onto glass coverslips in 24-well plates and allowed to adhere for 24 h before being treated with siRNA and/or drugs as indicated. Following siRNA/drug treatment, cells were permeablised in Triton-X100 for 1–3 min before being fixed in 4% PFA for 10 min. Cells were then blocked in 10% FCS for 1–2 h. Primary antibodies diluted in 2% FCS were added to each well and left overnight at 4 °C. The following day, cells were washed thoroughly with PBS before the secondary antibodies (AlexaFlour 488 or 594 made up at 1:1000 made in 2% FCS) were added for 1–2 h at room temperature in the dark. The cells were washed in PBS followed by a 5-min incubation in 1:1000 DAPI/PBS and then washed again in PBS alone before being mounted using immu-mount (Thermo, Waltham, MA, USA). Slides were imaged on a Nikon Eclipse TE200 Fluorescent Microscope and images were scored by counting the foci of ≈200 nuclei across five separate sections of the coverslip for each condition within an experimental repeat. For MGMT staining, cells were incubated with 2.5 M HCl for 30 min to denature the DNA prior to permeablisation. Primary antibodies used were 53BP (Abcam ab368823; 1:500), β-Actin (Abcam ab8226; 1:2000), β-Tubulin (Abcam ab7797; 1:2000), pDNA-PKcs ser2056 (Abcam ab18192; 1:1000), ERK5 (Santa Cruz sc-398015; 1:1000), FANCD2 (Abcam ab12450; 1:1000), MGMT (Abcam ab39253; 1:1000), O6-Methyl-2′-deoxyguanosine (Axxora SQX-SQM003.1; 1:100), RAD51 (Santa Cruz sc-8349; 1:500). Secondary antibodies used were all AlexaFlour 488 or 594 (both at 1:1000). COMET assays were carried out as previously described [45] using the Trevigen alkaline comet assay kit. In total, 50 cells per condition stained with SYBR Gold were imaged using the FITC channel and 20× lens on a Nikon Eclipse TE200 Fluorescent Microscope. Tail moment was calculated using TriTek Comet Score software as previously described [45].

### 4.5. Immunohistochemistry and Scoring

FFPE slides were submerged in xylene for 10 min followed by 100% ethanol for 10 min. Slides were then placed in 95% ethanol for 3 min followed by 30% hydrogen peroxide (in methanol) for 30 min. Antigen retrieval was carried out using 1:10 Dako Target Retrieval Solution (pH 6) in a pressure cooker for a 20-min heat cycle. Slides were then washed in PBS-t before being blocked in 10% goat serum made up in 1%BSA Triton-X100 in PBS for an hour. Anti-ERK5 antibody (Santa Cruz sc-398015) was diluted to 1:4000 in 2% goat serum and added to the slides overnight at 4 °C. Slides were washed in PBS-T before the secondary antibody (goat anti-mouse biotinylated, combined with streptavidin-HRP) was added to the slides 1:200 made up in 1% BSA in PBS-T for 1 h. The slides were washed with PBS-T and then Avidin-Biotin Complex (ABC) (Vector Laboratories) was added to the slides for 30 min. DAB substrate was then added to the slides for 30 s before quenching in water and the slides were then dehydrated in alcohol before being cleared in xylene and then mounted using DPX Mountant (Sigma, Kawasaki, Japan). TMAs (GL481, BS17016b and BS17016c) were purchased from US Biomax containing a range of normal and brain tumours across 88 cores, with an additional 102 astrocytoma samples retrieved from Histopathology at the Royal Hallamshire Hospital (ethics: YH-11-0319/STH15598). Overall, the 190 stained samples included 28 normal brain, 16 grade 1, 64 grade 2, 31 grade 3, and 51 grade 4 gliomas. ERK5 staining was scored by two independent scorers against negative control slides including by a consultant histopathologist using the scoring criteria; 0 (vasculature only due to the known expression of ERK5 within the vasculature), 1 (some but not all cells stained), 2 (low intensity pan stain), 3 (high intensity pan stain), which was the majority within the nucleus but also cytoplasmic.

### 4.6. FACS Analyses

At various times following siRNA and/or drug treatments, cell cultures were washed twice in PBS before being trypsinised and collected (both media and PBS washes were retained to collect any mitotic cells). The resulting cells were pelleted and washed in PBS before being processed using a FITC Annexin V apoptosis detection kit (BD Biosciences, San Jose, CA, USA, 556547) as described in the manufacture’s protocol. The resulting stained samples were detected on a FACSCalibur and subsequently analysed using FlowJo software to determine apoptotic and necrotic cell populations within each sample.

### 4.7. Cell Survival Assays

For MTT assays, untransfected or reversed transfected cells were plated at previously optimised/calibrated cell densities in 100 µL 10% FCS DMEM per well of a 96-well plate. Cells were incubated for 24 or 48 h prior to the addition of drugs at varying concentrations in DMSO as outlined in the figures. Five days after drug addition, 50 µL 3 mg/mL MTT diluted in PBS, was added to the cells and incubated for 3 h at 37 °C. Media was then removed and the resulting formazan precipitate dissolved in 200 µL DMSO. OD values were read at 540 nm on a Multiskan FC plate reader. The survival fraction was calculated compared to DMSO treated or DMSO treated and nontargeting siRNA transfected controls. For clonogenic survival assays, cells were seeded at previously optimised densities in 10 cm plates in 10 mL 10% FCS DMEM. After a 16-h incubation, various drugs were added in DMSO and cells cultured for 14–21 days. The media was then removed, and the resulting colonies were stained with 0.4% Methylene Blue for 30 min before rinsing. The colonies were counted and the survival fraction (SF) was calculated by the number of colonies counted/number of colonies plated multiplied by the plating efficiency (PE) derived from control/untreated plates.

## 5. Conclusions

Our findings provide the first validation of ERK5 as a potential therapeutic drug target to augment standard-of-care temozolomide treatment in high-grade adult brain tumours. Additionally, the data presented here add to the growing body of recent work that has shown that like its more established family members ERK1 and ERK2, as well as the wider MAP kinase signalling network, ERK5 has an impact on cancer biology and treatment response outside of its more established role in vascular stress. As such, our data together with that of others, provide a strong rationale for the further development of specific and potent small molecule ERK5 probes that could offer significant promise as future oncology therapeutics for these incurable cancers of unmet clinical need.

## Data Availability

The data presented in this study are available on request from the corresponding author. The data are not publicly available due to ethical considerations around patient material.

## Supplementary Materials

The following are available online at https://www.mdpi.com/2072-6694/13/5/944/s1. Figure S1. Identification and validation of ERK5 as a temozolomide sensitising factor, Figure S2. Target validation of ERK5 as a novel drug target in high grade gliomas.

## References

[1] Brain G.B.D. (2019). CNSCC Global, regional, and national burden of brain and other CNS cancer, 1990–2016: A systematic analysis for the Global Burden of Disease Study 2016. Lancet Neurol..

[2] Messaoudi K., Clavreul A., Lagarce F. (2015). Toward an effective strategy in glioblastoma treatment. Part I: Resistance mechanisms and strategies to overcome resistance of glioblastoma to temozolomide. Drug Discov. Today.

[3] Stupp R., Hegi M.E., Mason W.P., van den Bent M.J., Taphoorn M.J., Janzer R.C., Ludwin S.K., Allgeier A., Fisher B., Belanger K. (2009). Effects of radiotherapy with concomitant and adjuvant temozolomide versus radiotherapy alone on survival in glioblastoma in a randomised phase III study: 5-year analysis of the EORTC-NCIC trial. Lancet Oncol..

[4] Ostrom Q.T., Cote D.J., Ascha M., Kruchko C., Barnholtz-Sloan J.S. (2018). Adult Glioma Incidence and Survival by Race or Ethnicity in the United States From 2000 to 2014. JAMA Oncol..

[5] Aldape K., Brindle K.M., Chesler L., Chopra R., Gajjar A., Gilbert M.R., Gottardo N., Gutmann D.H., Hargrave D., Holland E.C. (2019). Challenges to curing primary brain tumours. Nat. Rev. Clin. Oncol..

[6] Jiang W., Jin G., Cai F., Chen X., Cao N., Zhang X., Liu J., Chen F., Wang F., Dong W. (2019). Extracellular signal-regulated kinase 5 increases radioresistance of lung cancer cells by enhancing the DNA damage response. Exp. Mol. Med..

[7] Pereira D.M., Gomes S.E., Borralho P.M., Rodrigues C.M.P. (2019). MEK5/ERK5 activation regulates colon cancer stem-like cell properties. Cell Death Discov..

[8] Simoes A.E., Rodrigues C.M., Borralho P.M. (2016). The MEK5/ERK5 signalling pathway in cancer: A promising novel therapeutic target. Drug Discov. Today.

[9] Pereira D.M., Rodrigues C.M.P. (2020). Targeted Avenues for Cancer Treatment: The MEK5-ERK5 Signaling Pathway. Trends Mol. Med..

[10] Stecca B., Rovida E. (2019). Impact of ERK5 on the Hallmarks of Cancer. Int. J. Mol. Sci..

[11] Finegan K.G., Perez-Madrigal D., Hitchin J.R., Davies C.C., Jordan A.M., Tournier C. (2015). ERK5 is a critical mediator of inflammation-driven cancer. Cancer Res..

[12] Kedika S.R., Shukla S.P., Udugamasooriya D.G. (2020). Design of a dual ERK5 kinase activation and autophosphorylation inhibitor to block cancer stem cell activity. Bioorg. Med. Chem. Lett..

[13] Lochhead P.A., Tucker J.A., Tatum N.J., Wang J., Oxley D., Kidger A.M., Johnson V.P., Cassidy M.A., Gray N.S., Noble M.E.M. (2020). Paradoxical activation of the protein kinase-transcription factor ERK5 by ERK5 kinase inhibitors. Nat. Commun..

[14] Wang G., Zhao Y., Liu Y., Sun D., Zhen Y., Liu J., Fu L., Zhang L., Ouyang L. (2020). Discovery of a Novel Dual-Target Inhibitor of ERK1 and ERK5 That Induces Regulated Cell Death to Overcome Compensatory Mechanism in Specific Tumor Types. J. Med. Chem..

[15] Nguyen D., Lemos C., Wortmann L., Eis K., Holton S.J., Boemer U., Moosmayer D., Eberspaecher U., Weiske J., Lechner C. (2019). Discovery and Characterization of the Potent and Highly Selective (Piperidin-4-yl)pyrido[3,2- d]pyrimidine Based in Vitro Probe BAY-885 for the Kinase ERK5. J. Med. Chem..

[16] Fleuren E.D., Zhang L., Wu J., Daly R.J. (2016). The kinome ‘at large’ in cancer. Nat. Rev. Cancer.

[17] Ferguson F.M., Gray N.S. (2018). Kinase inhibitors: The road ahead. Nat. Rev. Drug Discov..

[18] Broustas C.G., Duval A.J., Chaudhary K.R., Friedman R.A., Virk R.K., Lieberman H.B. (2020). Targeting MEK5 impairs nonhomologous end-joining repair and sensitizes prostate cancer to DNA damaging agents. Oncogene.

[19] Green D., Eyre H., Singh A., Taylor J.T., Chu J., Jeys L., Sumathi V., Coonar A., Rassl D., Babur M. (2020). Targeting the MAPK7/MMP9 axis for metastasis in primary bone cancer. Oncogene.

[20] Wu J., Cui H., Zhu Z., Wang L. (2016). MicroRNA-200b-3p suppresses epithelial-mesenchymal transition and inhibits tumor growth of glioma through down-regulation of ERK5. Biochem. Biophys. Res. Commun..

[21] Cook S.J., Tucker J.A., Lochhead P.A. (2020). Small molecule ERK5 kinase inhibitors paradoxically activate ERK5 signalling: Be careful what you wish for. Biochem. Soc. Trans..

[22] Hannus M., Beitzinger M., Engelmann J.C., Weickert M.T., Spang R., Hannus S., Meister G. (2014). siPools: Highly complex but accurately defined siRNA pools eliminate off-target effects. Nucleic Acids Res..

[23] Deng X., Elkins J.M., Zhang J., Yang Q., Erazo T., Gomez N., Choi H.G., Wang J., Dzamko N., Lee J.D. (2013). Structural determinants for ERK5 (MAPK7) and leucine rich repeat kinase 2 activities of benzo[e]pyrimido-[5,4-b]diazepine-6(11H)-ones. Eur. J. Med. Chem..

[24] Lin E.C., Amantea C.M., Nomanbhoy T.K., Weissig H., Ishiyama J., Hu Y., Sidique S., Li B., Kozarich J.W., Rosenblum J.S. (2016). ERK5 kinase activity is dispensable for cellular immune response and proliferation. Proc. Natl. Acad. Sci. USA.

[25] Bekker-Jensen S., Lukas C., Kitagawa R., Melander F., Kastan M.B., Bartek J., Lukas J. (2006). Spatial organization of the mammalian genome surveillance machinery in response to DNA strand breaks. J. Cell Biol..

[26] Shibata A., Jeggo P.A. (2020). Roles for 53BP1 in the repair of radiation-induced DNA double strand breaks. DNA Repair.

[27] Petermann E., Orta M.L., Issaeva N., Schultz N., Helleday T. (2010). Hydroxyurea-stalled replication forks become progressively inactivated and require two different RAD51-mediated pathways for restart and repair. Mol. Cell.

[28] Yajima H., Lee K.J., Chen B.P. (2006). ATR-dependent phosphorylation of DNA-dependent protein kinase catalytic subunit in response to UV-induced replication stress. Mol. Cell. Biol..

[29] Gil Del Alcazar C.R., Todorova P.K., Habib A.A., Mukherjee B., Burma S. (2016). Augmented HR Repair Mediates Acquired Temozolomide Resistance in Glioblastoma. Mol. Cancer Res..

[30] Cicenas J., Kalyan K., Sorokinas A., Stankunas E., Levy J., Meskinyte I., Stankevicius V., Kaupinis A., Valius M. (2015). Roscovitine in cancer and other diseases. Ann. Transl. Med..

[31] Collis S.J., Barber L.J., Clark A.J., Martin J.S., Ward J.D., Boulton S.J. (2007). HCLK2 is essential for the mammalian S-phase checkpoint and impacts on Chk1 stability. Nat. Cell Biol..

[32] Myers K.N., Barone G., Ganesh A., Staples C.J., Howard A.E., Beveridge R.D., Maslen S., Skehel J.M., Collis S.J. (2016). The bornavirus-derived human protein EBLN1 promotes efficient cell cycle transit, microtubule organisation and genome stability. Sci. Rep..

[33] Zieve G.W., Turnbull D., Mullins J.M., McIntosh J.R. (1980). Production of large numbers of mitotic mammalian cells by use of the reversible microtubule inhibitor nocodazole. Nocodazole accumulated mitotic cells. Exp. Cell Res..

[34] Aparicio T., Baer R., Gautier J. (2014). DNA double-strand break repair pathway choice and cancer. DNA Repair.

[35] Bonetti D., Colombo C.V., Clerici M., Longhese M.P. (2018). Processing of DNA Ends in the Maintenance of Genome Stability. Front. Genet..

[36] Scully R., Panday A., Elango R., Willis N.A. (2019). DNA double-strand break repair-pathway choice in somatic mammalian cells. Nat. Rev. Mol. Cell Biol..

[37] Gusev Y., Bhuvaneshwar K., Song L., Zenklusen J.C., Fine H., Madhavan S. (2018). The REMBRANDT study, a large collection of genomic data from brain cancer patients. Sci. Data.

[38] Patil A.A., Sayal P., Depondt M.L., Beveridge R.D., Roylance A., Kriplani D.H., Myers K.N., Cox A., Jellinek D., Fernando M. (2014). FANCD2 re-expression is associated with glioma grade and chemical inhibition of the Fanconi Anaemia pathway sensitises gliomas to chemotherapeutic agents. Oncotarget.

[39] Koncar R.F., Dey B.R., Stanton A.J., Agrawal N., Wassell M.L., McCarl L.H., Locke A.L., Sanders L., Morozova-Vaske O., Myers M.I. (2019). Identification of Novel RAS Signaling Therapeutic Vulnerabilities in Diffuse Intrinsic Pontine Gliomas. Cancer Res..

[40] Roskoski R. (2018). Targeting oncogenic Raf protein-serine/threonine kinases in human cancers. Pharmacol. Res..

[41] Svilar D., Dyavaiah M., Brown A.R., Tang J.B., Li J., McDonald P.R., Shun T.Y., Braganza A., Wang X.H., Maniar S. (2012). Alkylation sensitivity screens reveal a conserved cross-species functionome. Mol. Cancer Res..

[42] Thaker N.G., Zhang F., McDonald P.R., Shun T.Y., Lewen M.D., Pollack I.F., Lazo J.S. (2009). Identification of survival genes in human glioblastoma cells by small interfering RNA screening. Mol. Pharmacol..

[43] Caragher S., Chalmers A.J., Gomez-Roman N. (2019). Glioblastoma’s Next Top Model: Novel Culture Systems for Brain Cancer Radiotherapy Research. Cancers.

[44] Rominiyi O., Al-Tamimi Y., Collis S.J. (2019). The ‘Ins and Outs’ of Early Preclinical Models for Brain Tumor Research: Are They Valuable and Have We Been Doing It Wrong?. Cancers.

[45] Staples C.J., Barone G., Myers K.N., Ganesh A., Gibbs-Seymour I., Patil A.A., Beveridge R.D., Daye C., Beniston R., Maslen S. (2016). MRNIP/C5orf45 Interacts with the MRN Complex and Contributes to the DNA Damage Response. Cell Rep..

