# Supplementary material for: Identification and Validation of ERK5 as a DNA Damage Modulating Drug Target in Glioblastoma
Source: Cancers (Basel). 2021 Feb 24;13(5):944. doi: 10.3390/cancers13050944 (PMC7956595; doi:10.3390/cancers13050944)
Supplement: Supplementary file 1 [file cancers-13-00944-s001.zip › Cancers-1110277_supplementary_conversion/cancers-1110277_supplementary_conversion.docx]

Supplementary Materail: Identification and Validation of ERK5 as a DNA Damage Modulating Drug Target in Glioblastoma

Natasha Carmell ^1^, Ola Rominiyi ^1,2^, Katie N. Myers ^1^, Connor McGarrity-Cottrell ^1^, Aurelie Vanderlinden ^1^, Nikita Lad ^1^, Eva Perroux-David ^1^, Sherif F. El-Khamisy ^3,4^, Malee Fernando ^5^, Katherine G. Finegan ^6^, Stephen Brown ^7^ and Spencer J. Collis ^1,3,^*


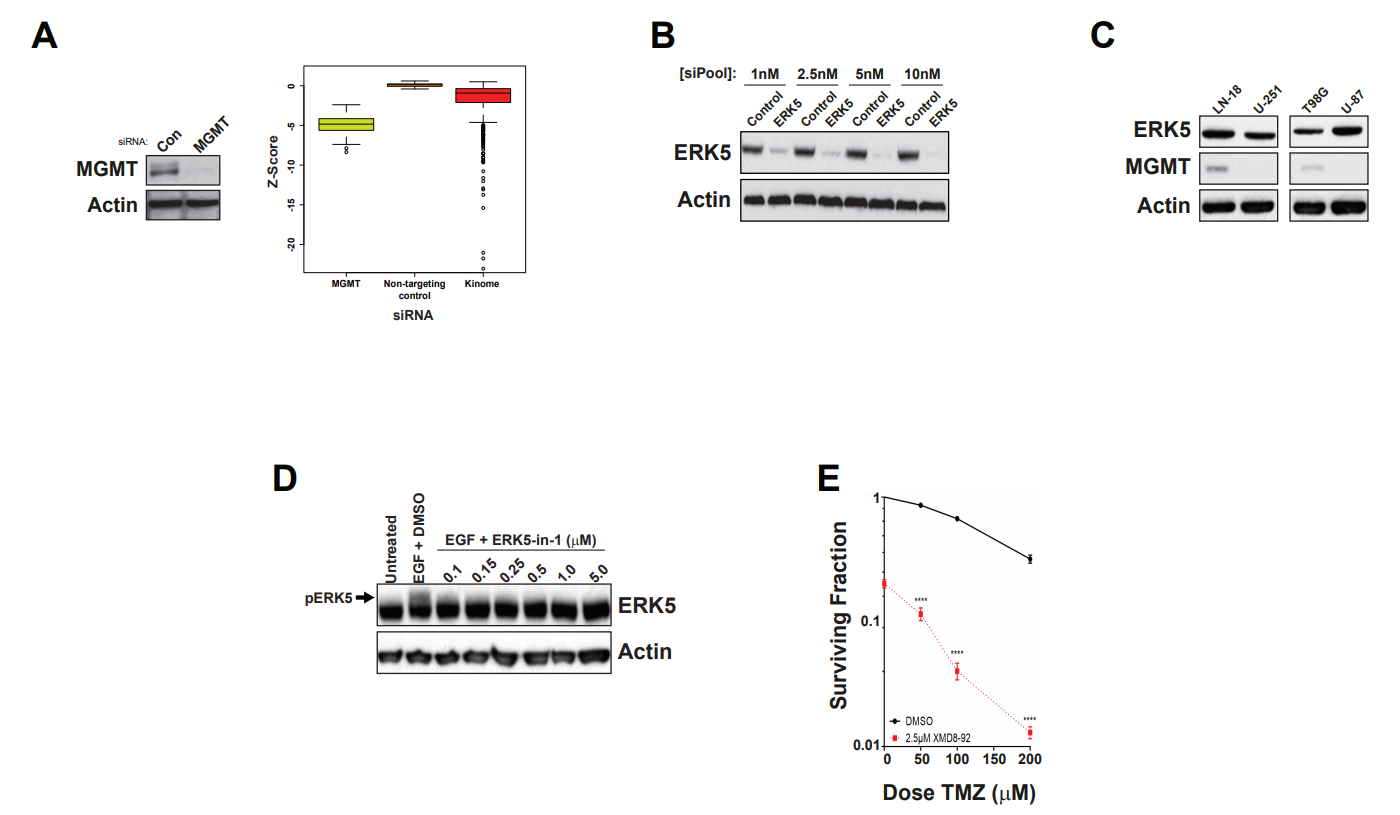


**Figure 1.** Identification and validation of ERK5 as a temozolomide sensitising factor. **A:** Left panel; representative Western blot of MGMT siRNA knockdown within these cells. Right panel; graphical representation of the rank plot summary of the kinome-targeted siRNA representing TMZ sensitivity compared with cells transfected with either nontargeting siRNA (-ve control) or those transfected with MGMT siRNA (+ve control). **B:** Western blots for the indicated proteins in T98G cells showing optimisation of ERK5 targeting siPOOLs. **C:** Western blots for the indicated proteins in the indicated glioblastoma cells showing ERK5 protein expression levels. MGMT status for these cells is also confirmed within these blots. **D:** Western blots for the indicated proteins in T98G cells showing EGF-stimulated ERK5 activation as visualised by the appearance of a higher molecular weight phosphorylated form of ERK5 (black arrow), and subsequent inhibition following 90min pretreatment with the indicated dose of ERK5-in-1. **E:** TMZ cytotoxicity as assessed by clonogenic survival assay in LN18 cells pretreated with either DMSO or 2.5μM of the small molecule ERK5 inhibitor XMD-82. Statistical significance was calculated using the nonparametric Mann–Whitney U-test: ns = not significant, * = *p* < 0.05, * = *p* < 0.01, *** = *p* < 0.001, and **** = *p* < 0.0001 comparing the indicated treatment to DMSO controls or to another indicated treatment cell population.


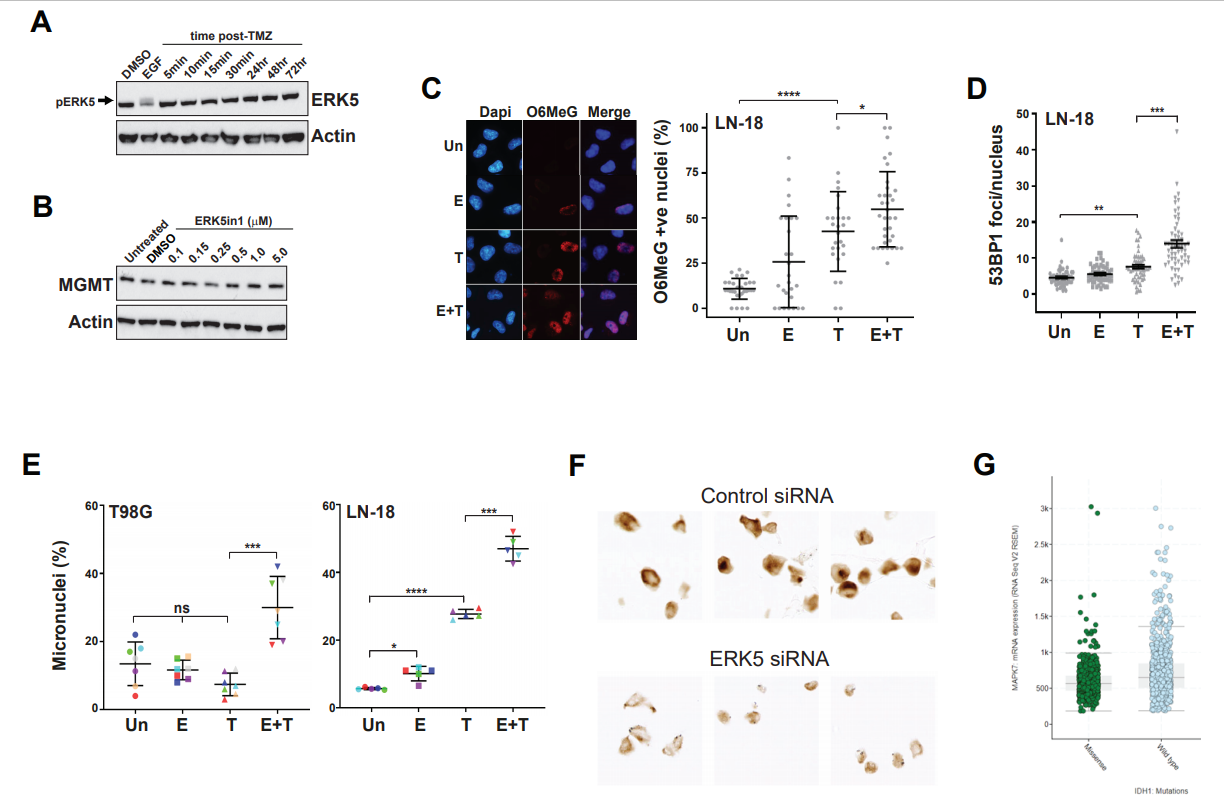


**Figure 2.** Target validation of ERK5 as a novel drug target in high grade gliomas. **A:** Western blots for the ERK5 and actin in T98G cells showing EGF-stimulated ERK5 phosphorylation (black arrow) that is not observed following TMZ treatment for the indicated time period. **B:** Western blots for MGMT and actin in T98G cells following 90min treatment with the indicated dose of the ERK5-in-1 compound. **C:** Representative immunofluorescence images (left panels) and associated scoring (right panels) of O^6^MeG lesions in LN18 cells 24h following treatment with either DMSO, 250nM of ERK5-in-1 (E), 200μM TMZ (T), or a combination of the two treatments (E + T). D: Scoring of 53BP1 nuclear foci in LN-18 cells 24h following treatment with either DMSO, 250nM of ERK5-in-1 (E), 200μM TMZ (T), or a combination of the two treatments (E + T). **E:** Scoring of micronuclei in T98G and LN-18 cells 24h following treatment with either DMSO, 250nM of ERK5-in-1 (E), 200μM TMZ (T), or a combination of the two treatments (E + T). **F:** Representative bright field images of immunohistochemical staining in FFPE T98G cells transfected with either nontargeting control or previously validated ERK5 siRNA as indicated. G: Graph showing relative ERK5 mRNA expression levels in either WT or IDH1 mutant gliomas. Data shown was derived from over 5900 gliomas within multiple independent clinical cohorts (cBioPortal). Statistical significance was calculated using the nonparametric Mann–Whitney U-test: ns = not significant, * = *p* < 0.05, * = *p* < 0.01, *** = *p* < 0.001, and **** = *p* < 0.0001 comparing the indicated treatment to DMSO controls or to another indicated treatment cell population.
