# Supplementary material for: Identification and Validation of ERK5 as a DNA Damage Modulating Drug Target in Glioblastoma
Source: Cancers (Basel). 2021 Feb 24;13(5):944. doi: 10.3390/cancers13050944 (PMC7956595; doi:10.3390/cancers13050944)
Supplement: Supplementary file 1 [file cancers-13-00944-s001.zip › Cancers-1110277_supplementary_conversion/cancers-1110277_WB data.pdf]

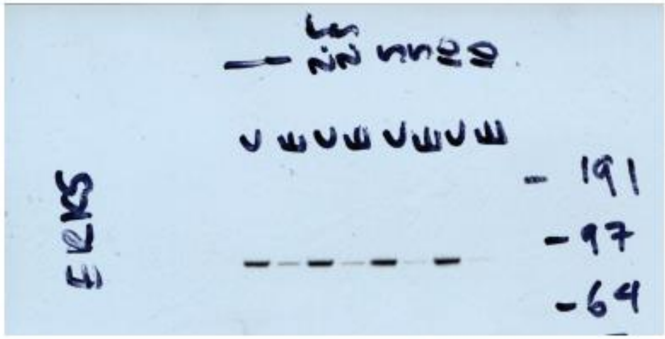

3789.941
849.456
4166.527
621.456
4370.820
364.920
4269.406
180.849

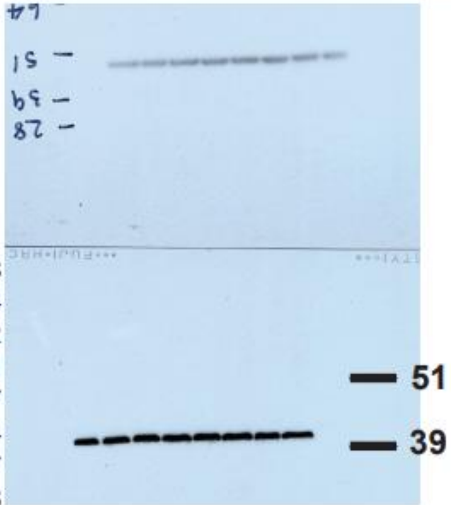

4189.648
5301.184
5242.062
5418.891
5689.477
5827.184
5379.477
4831.648

Figure S1B (ERK5 and actin)

7854.205
8449.205
11468.841
8605.376
9235.619
7935.497

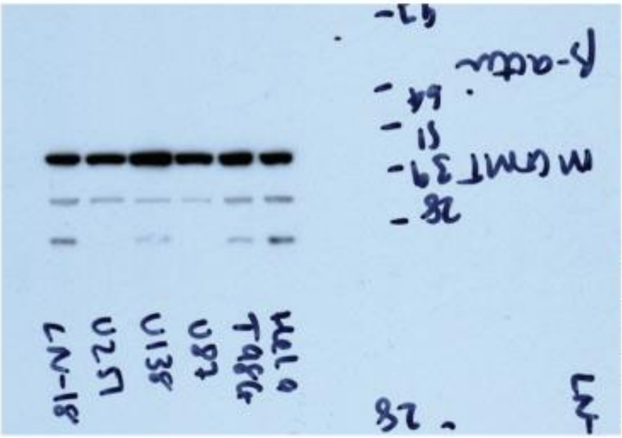

4454.305
461.607
1425.062
16.536
1521.820
5164.548

Figure S1C (MGMT and actin)

8676.711
8961.075
10122.196
9671.782
10485.196
9742.660
10010.075
10734.489
11430.660

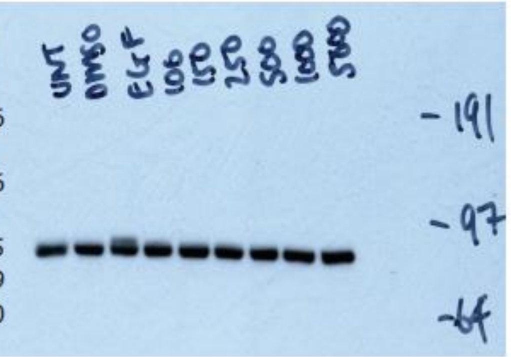

10388.539
8144.296
8889.953
8657.125
8355.832
9958.196
9122.418
8226.711
11249.004

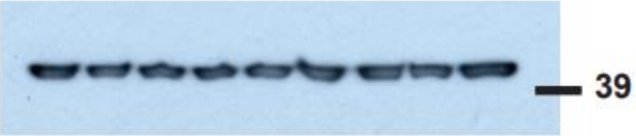

Figure S1D (pERK5/ERK5 and actin)

7237.225
6735.225
5325.447
5775.276
5893.861
4503.205
5436.569
6004.276
6383.154

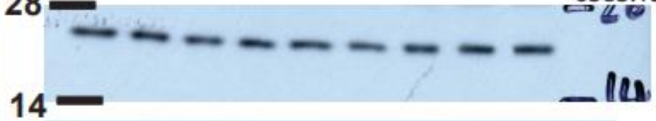

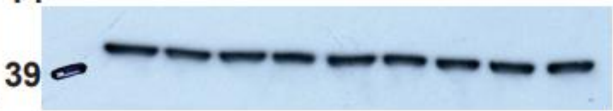

Figure S2B (MGMT and actin)

8654.468
7364.468
7736.761
7620.761
8440.175
7690.933
7695.225
7843.690
8232.690

Gel images and densitometry quantification for (Carmell et al)
